# Alcoholism Treatment and Total Health Care Utilization and Costs

**Published:** 1995

**Authors:** Anne Geller

**Affiliations:** Anne Geller, M.D., is the chief of the Smithers Alcoholism Treatment and Training Center, St. Lukes–Roosevelt Hospital Center, New York, New York

**Keywords:** AOD dependence, treatment cost, treatment outcome, cost-effectiveness

**Figure:**
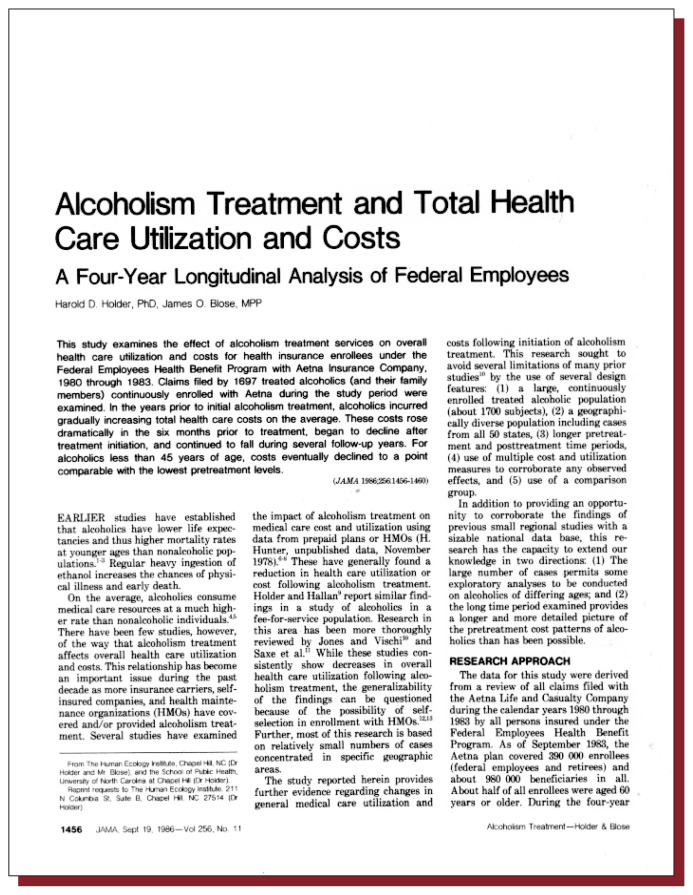
Holder, H.D., and Blose, J.O. Alcoholism treatment and total health care utilization and costs: A four-year longitudinal analysis of Federal employees. *Journal of the American Medical Association* 256(11):1456–1460, 1986.

This study by Holder and Blose attempted to answer an important health policy question: Can alcoholism treatment, as opposed to treating alcohol-related medical complications—because it is actually provided to a large population motivated to seek care—result in reduced overall health care costs? Few studies had been done to examine how alcoholism treatment affected overall health care utilization and costs, and as more insurance companies and health maintenance organizations (HMO’s) began covering or providing alcoholism treatment, this became an important question. The results of Holder and Blose’s study provided significant evidence that alcoholism treatment really was cost-effective for health care plans.

Holder and Blose’s study was the first to examine alcoholism treatment and health care costs in a large (approximately 1,700 persons) continuously enrolled treated population over a long (4 year) time period, which gave the researchers time to study the population for several months both before and after treatment. The population was geographically diverse, coming from all 50 States, and multiple cost-and-utilization measures were used to determine treatment effectiveness. The size of the sample permitted analysis on alcoholics of different ages, and the longer time period made possible a more detailed view of alcoholics’ health care pre-treatment cost patterns. Holder and Blose’s study also was the first to show the economic advantage of making treatment available in the early stages of alcoholism and thus provided a rationale for identification and intervention programs to motivate alcoholics to be treated at a younger age and earlier in the course of their disease.

Previous studies, using data from HMO’s or prepaid plans and fee-for-service systems, that also had indicated reduced health care costs as a result of providing alcoholism treatment had been plagued by methodological problems, such as small numbers of cases in specific geographic areas. These problems had severely limited their generalizability and thus their usefulness for health policy.

Holder and Blose’s study looked at health care utilization and costs for enrollees under the Federal Employees Health Benefits Program with Aetna insurance company. Alcoholics were defined as any person receiving treatment under the primary diagnosis of alcoholism. Claims filed by 1,697 treated alcoholics and their family members continuously enrolled during the study period were examined together with a sample of 3,598 randomly selected, similarly aged, enrolled families with no family members receiving alcoholism treatment. The 4-year average per capita monthly pretreatment health care costs for a family with an alcoholic member was almost 100 percent ($209.60 compared with $106.50) above that for a family with no members receiving alcoholism treatment for the same period. (Much of this cost came from inpatient alcoholism treatment; however, even without these treatment expenses, the average per capita monthly health care costs for families with alcoholic members were still higher: $180.88).

The average monthly health care costs for alcoholics gradually increased over the 36 months prior to treatment, rising dramatically in the last 6 months. Holder and Blose interpreted this to mean that the average alcoholic’s emotional and physical problems appeared to increase in the 6 months before he or she decided to enter treatment. After alcoholism treatment, costs for the alcoholic population declined and continued to do so for the next few years. This pattern was almost identical for men and women.

For those alcoholics who were 44 years of age and under at the time of treatment, posttreatment health care costs eventually fell to those seen 36 months prior to treatment. For those 65 years of age and over, the posttreatment decline in costs was not so dramatic, and health care costs remained above pretreatment levels. Holder and Blose suggested that costs in this older population could be attributed to increasing medical care costs caused by aging as well as to the existence of alcohol-related health problems resulting from a longer period of abuse.

Subsequent studies, many also by Holder and Blose, have confirmed and extended these original findings. For example, Holder and colleagues (1992) reviewed a 20-year period of research into the potential total health care cost savings associated with alcoholism treatment, further confirming that untreated alcoholics use health care resources at twice the rate of their population control group and that this difference can be eliminated if they undergo alcoholism treatment. This study supported the results of the original Holder and Blose study showing that younger drinkers, if treated for their alcoholism, will have lower health care costs than older nontreated alcoholics.

Although Holder and Blose’s study was not designed to compare treatments, treatment costs, and lengths of stay, their finding that overall treatment for alcoholism reduced subsequent health care costs encouraged further research examining the effectiveness and cost-effectiveness of various types of treatment. For example, Holder and colleagues (1991) found that overall, the most effective treatment modalities were not the most expensive. This finding should further stimulate researchers to conduct clinical studies in which both cost and effectiveness are measured.

It is now clear that the future of alcoholism treatment depends on extending cost-effectiveness studies beyond those of generic “alcoholics” to identifying different types of alcohol problems (e.g., alcohol abuse, alcohol dependence) and beyond generic “treatment” to more specific treatment techniques or settings (e.g., inpatient, intensive outpatient). Once these distinctions are made, studies can be done on the most cost-effective type of treatment for each type of alcohol problem. Holder and Blose’s landmark study demonstrated elegantly and definitively the cost benefits of any type of alcoholism treatment. They also proved that research in the alcoholism treatment field could yield results that were as exact as that of research in other branches of medicine.
